# Surveillance of West Nile virus infection in Kashgar Region, Xinjiang, China, 2013–2016

**DOI:** 10.1038/s41598-021-93309-2

**Published:** 2021-07-07

**Authors:** Yanping Zhang, Wenwen Lei, Yali Wang, Haitian Sui, Bo Liu, Fan Li, Ying He, Zhaoxia Li, Shihong Fu, Lu Wang, Limin Xu, Muti Mahe, Zhenguo Gao, Tuerxun Mamutijiang, Zhi Lv, Nijuan Xiang, Lei Zhou, Daxin Ni, Guodong Liang, Qun Li, Huanyu Wang, Zijian Feng

**Affiliations:** 1 Chinese Center for Disease Control and Prevention, Beijing, 102206 People’s Republic of China; 2 Department of Viral Encephalitis, NHC Key Laboratory of Biosafety, National Institute for Viral Disease Control and Prevention, State Key Laboratory of Infectious Disease Prevention and Control, Chinese Center for Disease Control and Prevention, Beijing, 102206 People’s Republic of China; 3 China National Biotec Group Company Limited, Beijing, 100024 People’s Republic of China; 4 Center for Drug Evaluation of the China National Medical Products Administration, Beijing, 100022 People’s Republic of China; 5 Kashgar Center for Disease Control and Prevention of Xinjiang, Kashgar, 844000 People’s Republic of China; 6 Xinjiang Uygur Autonomous Region Center for Disease Control and Prevention, Urumqi, 830001 People’s Republic of China; 7 Jiashi Center for Disease Control and Prevention, Jiashi, 844300 People’s Republic of China

**Keywords:** Infectious-disease diagnostics, Virology

## Abstract

West Nile virus (WNV) was first isolated in mainland China from mosquitoes in Jiashi County, Kashgar Region, Xinjiang in 2011, following local outbreaks of viral meningitis and encephalitis caused by WNV. To elaborate the epidemiological characteristics of the WNV, surveillance of WNV infection in Kashgar Region, Xinjiang from 2013 to 2016 were carried out. Blood and CSF samples from surveillance human cases, blood of domestic chicken, cattle, sheep and mosquitoes in Kashgar Region were collected and detected. There were human 65 WNV Immunoglobulin M (IgM) antibody positive cases by ELISA screening, 6 confirmed WNV cases by the plaque reduction neutralization test (PRNT) screening. These cases occurred mainly concentrated in August to September of each year, and most of them were males. WNV-neutralizing antibodies were detected in both chickens and sheep, and the positive rates of neutralizing antibodies were 15.5% and 1.78%, respectively. A total of 15,637 mosquitoes were collected in 2013–2016, with *Culex pipiens* as the dominant mosquito species. Four and 1 WNV-positive mosquito pools were detected by RT-qPCR in 2013 and 2016 respectively. From these data, we can confirm that Jiashi County may be a natural epidemic foci of WNV disease, the trend highlights the routine virology surveillance in WNV surveillance cases, mosquitoes and avian should be maintained and enhanced to provide to prediction and early warning of outbreak an epidemic of WNV in China.

## Introduction

West Nile virus (WNV) (family Flaviviridae, genus Flavivirus) is a zoonotic mosquito-borne flavivirus that causes severe illness and death in horses, humans, and birds. It has a complex life cycle involving several bird species as the primary host, mosquitoes as the primary vector and humans, and horses as incidental or dead-end hosts^1,2^. In humans, approximately 80% of WNV infections are asymptomatic, while 20% cause mild hyperpyrexia, known as West Nile fever (WNF). However, approximately 1% of cases are more severe, resulting in West Nile Neuroinvasive Disease (WNND)^3^.

In recent years, as a reemerging infectious disease, WNV is expanding its geographic range in Europe as well as in other parts of the world, resulting in an increased number of disease outbreaks associated with human morbidity and mortality^4–6^. According to WHO, WNV is now endemic mainly in Southern and Eastern Europe, Africa, North America, Western Asia, the Middle East and Australia^7^. In 2018, 2083 cases of WNV and 181 deaths have been recorded in Europe, which shows a significant increase of WNV cases compared with the 2017 transmission season^8^.

The first virologically confirmed WNV infections and first isolation from mosquito specimens collected in Jiashi County, Kashgar Region, Xinjiang of western China were reported for 2011^9^. The prevalence of fever and outbreaks of viral encephalitis caused by WNV infection has been recorded at a local scale^10–12^, demonstrating the circulation of WNV and its prevalence in Xinjiang, China. Xinjiang, located in the northwestern border of China, is the largest provincial-level administrative region in China, which has a long borderline with eight adjoining countries. According to reports, there are WNV case and outbreaks in India^13^, Russia^14^ and Pakistan^15^ bordering Xinjiang. Therefore, monitoring WNV infection in this region is important for public health. The analysis of WNV in mosquitoes as well as geographic information about acute WNV infection in Xinjiang and exposure of the general population can provide valuable information about WNV transmission not only in China but also in neighboring countries. Here, we present the comprehensive results of human–animal–vector surveillance of WNV infection in Xinjiang from 2013 to 2016.

## Material and methods

### Human samples

#### Surveillance case definitions for WNF and WNND

The probable and confirmed case definitions for WNF and WNND were approved by the Chinese Center for Disease Control and Prevention (CCDC) in 2013. A case must meet at least one of the clinical criteria and at least one of the laboratory criteria described in the following sections.

#### Clinical criteria for diagnosis^16^

WNF requires, at a minimum, the presence of fever, as measured by the patient or clinician, the absence of neuroinvasive disease, and the absence of a more likely clinical explanation for the illness.

WNND requires the presence of fever and at least one of the following, as documented by a physician and in the absence of a more likely clinical explanation: (1) acutely altered mental status (e.g., disorientation, obtundation, stupor, or coma), (2) other acute signs of central or peripheral neurologic dysfunction (e.g., paresis or paralysis, nerve palsies, sensory deficits, abnormal reflexes, generalized convulsions, or abnormal movements), or (3) pleocytosis (increased white blood cell concentration in cerebrospinal fluid (CSF)) associated with illness clinically compatible with meningitis (e.g., headache or stiff neck).

Acute flaccid paralysis (AFP) requires the presence of fever, asymmetry deep sputum emission slowed or disappeared, muscle nerve showed demyelinating changes, facial nerve palsy, severe muscle weakness (bilateral or unilateral upper limb muscle weakness progressively developed, lower limbs are weak or even paralyzed). And cannot be explained by other reasons.

#### Laboratory criteria for diagnosis

For a case to be considered laboratory confirmed, at least one of the following criteria should be met: (1) isolation of virus from blood or CSF; or (2) detection of genomic sequences in blood or CSF; or (3) virus-specific immunoglobulin M (IgM) antibodies demonstrated in CSF by Enzyme-linked Immunosorbent Assay (ELISA); or (4) a fourfold or greater change in virus-specific serum antibody titer.

Probable cases have virus-specific serum IgM antibodies detected by ELISA, but with no available results of a confirmatory test for virus-specific serum IgG antibodies in the same or a later specimen.

#### Sample collection

Blood and CSF samples from patients with WNV disease were collected during the WNV transmission seasons from July to October 2013, to 2016. All samples originate from routine surveillance activities of the corresponding institute. Case monitoring of WNND in the First People's Hospital of Kashgar and the Second People's Hospital of Kashgar was conducted. In addition, the Jiashi County People's Hospital and five village hospitals simultaneously monitored cases of WNF and WNND. The monitoring departments were mainly fever clinics, emergency departments, internal medicine, pediatrics and other departments (including outpatient department and wards).

For patients with WNF, collect acute blood (3–5 mL) specimens were collected and stored them at − 20℃ after separation of serum. For patients with WNND, collect blood (3–5 mL) and CSF (2 mL) specimens were collected. For patients with positive WNV-IgM antibody in the acute phase, recovery blood (3–5 mL) was collected at the third week of onset, and serum was separated and stored at − 20 °C. All samples were first sent to Kashgar Center for Disease Control and Prevention (CDC) for WNV IgM test and then sent to China CDC for confirmation.

### Animal samples

In 2013, before the start of the monitoring work in Jiashi County, 200 chickens were selected and dispersed throughout the farmhouse. These chickens had originated from disease-free conditions. From May to the end of October, the blood samples (2 mL) were collected once every ten days to test for WNV IgM antibody.

Between August and September in 2015, 167 animal (166 sheep and 1 cattle) blood samples (2 mL) were collected from the slaughterhouses in Kashgar City, Kashgar Region, for WNV IgM antibody detection.

### Mosquito collection

Mosquito sampling was performed at different sites in Jiashi County according to previously described methods^17^. Sampling was conducted from July to September 2013–2016, with the exception that no sampling was conducted in 2014. Ten gravid traps were placed at ten sampling sites and operated overnight (from 19:00 to 08:00). Epidemiological characteristics were considered for the sampling site selection including occurrence of WNV human cases, proximity to wetlands and migratory bird resting or stopover sites. Mosquito traps were placed in houses and livestock farms.

Field-collected specimens were transported to the laboratory, and female mosquitoes were pooled by date and collection site by handling mosquitoes individually with sterile stainless steel tweezers with a maximum of 50 individuals per pool and stored in cryotubes at − 70 °C until being assayed for viral detection.

### Laboratory detection

All of the human serum and CSF samples underwent ELISA detection of WNV IgM antibodies. The WNV-IgM Test Kit (WNV IgM Capture DxSelect ELISA; Focus) employed the Capture ELISA procedure^11,18^. All IgM antibody positive samples were subjected to plaque reduction neutralization test (PRNT). In order to avoid serum cross-positive, both Japanese encephalitis virus (JEV) and WNV were detected simultaneously. In PRNT, WNV XJ11129 strain (GenBank: JX442279, isolated and preserved in our laboratory), JEV P3 strain (GenBank: JEU47032, preserved in our laboratory) and Vero cells (African green monkey kidney cells, preserved in our laboratory) were employed and the test was performed as previously described with minor modifications^12^. Briefly, the serum was inactivated at 56 °C for 30 min. The inactivated sera were diluted at 1:10, 1:20, 1:40, 1:80, 1:160, 1:320, 1:640, and 1:1280 respectively, and mixed with diluted JEV/WNV (200 PFU) in an equal volume; meanwhile, 100 PFU, 10 PFU and 1 PFU were used as the titration of viral challenge dose of virus diluent, and MEM culture medium was mixed with an equal volume of 200 PFU of virus as the cell blank control solution. The mixture was added to six-well Vero cells and incubated in a 5% CO_2_ incubator at 37 °C for 60 min, followed by overlaying with 1.1% methylcellulose–MEM culture medium for 5 days. Crystalline violet staining was used to calculate the number of plaques per well. The maximum serum dilution with 90% plaque reduction was used as the serum neutralizing antibody titer^10,11^. If the virus neutralizing antibody titer is ≥ 1:10 in the plaque reduction neutralization test result, it is judged as neutralizing antibody positive. If the PRNT90 anti-WNV antibody titer was at least fourfold greater than that of the corresponding JEV antibody titer in the same sample, it was identified as WNV infection rather than JEV infection. Otherwise, it was identified as JEV infection^10,11^.

WNV exposure was evaluated in sera obtained from chickens, cattle and sheep from Jiashi County via the detection of specific neutralizing antibodies by PRNT. The animal serum was heat inactivated and diluted to 1:10 to detect WNV-neutralizing antibodies. The rest of the steps are the same as the human detection method. Specimens were judged positive when PRNT90-neutralizing antibody was ≥ 1:10.

Mosquito pools were homogenised in sterile 1 mL phosphate-buffered saline, viral RNA was extracted from 140 μL of supernatants using the QIAamp Viral RNA Extraction Kit (QIAGEN Inc., Valencia, CA) according to the manufacturer’s instructions. All nucleic acid extracts were screened by a published WNV (lineage 1 + 2) RT-qPCR^19^, using reagents of the AgPath-ID One-Step RT-PCR Kit (Life Technologies, ABI Ambion, USA). Positive samples were subsequently investigated by various conventional RT-PCRs targeting the complete WNV genomes by employing published primer pairs^20,21^. The RT-PCR assays, sequencing reactions and sequence alignments were performed as described previously^19^.

### Ethical statement

This study was approved by the Ethics Committee of the Chinese Center for Disease Control and Prevention (CCDC). The implementation agencies of this study were the National Reference Lab of JEV and the WHO-JE-Reference Lab-China, responsible for the surveillance of JE and WN in mainland China. All experiments were performed in accordance with relevant guidelines and regulations. And all methods were performed in accordance with the relevant guidelines and regulations for human studies. Research carried out on humans was in compliance with the Helsinki Declaration. The Ethics Committee of the Chinese Center for Disease Control and Prevention (CCDC) approved this study and waived the requirement for written informed consent. All animal experiments were carried out in accordance with the guidelines of the Beijing Municipality on the Review of Welfare and Ethics of Laboratory Animals approved by the Beijing Municipality Administration Office of Laboratory Animals (BAOLA). No animals were killed for the purpose of this study. All animals were not sacrificed and given certain economic compensation to the animal owners. The study was carried out in compliance with the ARRIVE guidelines and the experiment reporting adheres to the ARRIVE guidelines. The data elements collected for these monitoring activities represent the minimum necessary interference. All human and animal data were reported to CCDC, which extended the report to the National Health Commission.

## Results

### Human infection

A total of 3053 acute phase serum samples of monitoring WNF and WNND cases and 313 CSF samples of monitoring WNND cases were collected from 2013 to 2016. All CSF samples were negative for IgM antibody by ELISA and 65 serum samples were positive for WNV IgM antibody (Table 1). Of the 65 cases, convalescent sera were collected in 41 cases, of which 6 fever cases had a ≥ 4-fold difference in WNV-neutralizing antibody titer between the convalescent/acute serum samples were identified as being positive for WNV infection. Meanwhile, the titer of the WNV-neutralizing antibody was higher than that of the JEV PRNT90 antibody (Table 2). These six cases are concentrated in 2013. According to the clinical and laboratory criteria for diagnosis, these six cases were confirmed as WNF cases.

**Table 1:** Surveillance cases of WNV disease in Kashgar Region, 2013–2016.

Year	WNF surveillance cases			WNND surveillance cases				
	Number of cases	Acute serum	WNV-IgM positive	Number of cases	Acute serum	CSF	Serum WNV-IgM positive	CSF WNV-IgM positive
2013	683	683	42 (0.014)	9	9	0	9 (0.003)	0
2014	473	473	2 (0.0006)	77	66	11	0	0
2015	863	863	0	176	52	111	1 (0.0003)	0
2016	857	857	11 (0.004)	191	50	191	0	0
Total	2876	2876	55	383	177	313	10	0

**Table 2:** Detection for WNV infection in Kashgar Region, 2013.

Number	WNV IgM-ELISA	WNV PRNT90		JEV IgM-ELISA	JEV PRNT90	
		Acute serum	Convalescent serum		Acute serum	Convalescent serum
1	+	1:80	1:320	+	1:10	1:20
2	+	< 1:10	1:40	+	< 1:10	< 1:10
3	+	1:10	1:40	−	< 1:10	< 1:10
4	+	1:20	1:80	−	< 1:10	1:10
5	+	< 1:10	1:160	+	< 1:10	< 1:10
6	+	< 1:10	1:40	−	< 1:10	< 1:10

**Figure 1:**
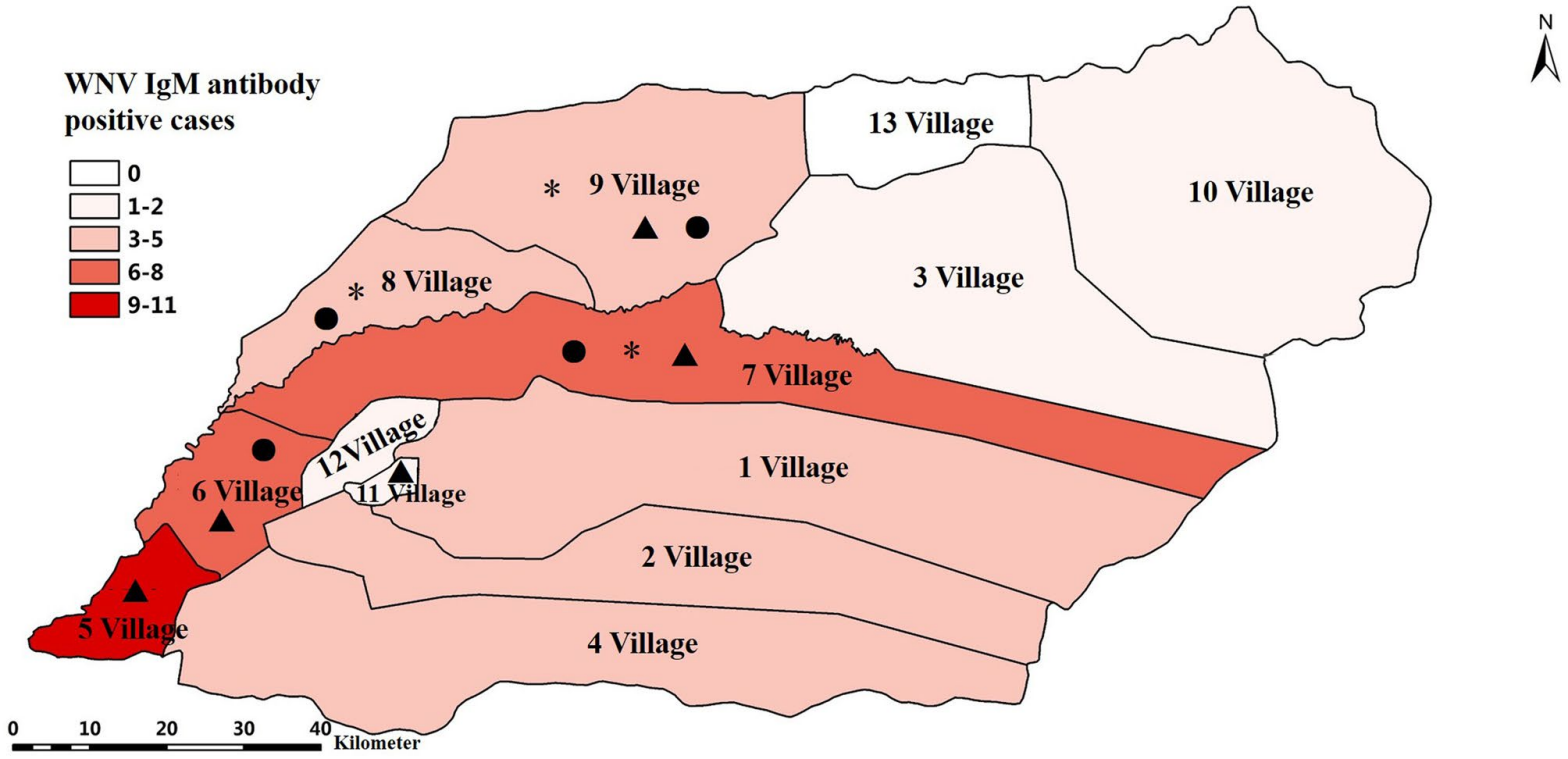
Distribution of human and animal WNV cases in Jiashi County, Kashgar Region, Xinjiang, China. (2013–2016). The map in figure was created using ArcGIS 10.6 software (ESRI, Redlands, USA). “▲” represents the confirmed WNF cases; “●” represents the locations where the chicken WNV neutralizing antibody is positive; “*” represents the sampling locations of the WNV positive mosquitoes.

Sixty-five IgM antibody-positive cases were widely distributed in 11 villages in Jiashi County (Fig. 1), and no clustered cases were found. The annual onset was from August to October, showing a distinct seasonal onset, with the highest number of cases (23 cases) in late August, accounting for 35% of all cases. Male cases were predominant (40/65, 61.5%), and there were WNV IgM antibody positive cases in all age groups.

Six WNF cases lived in 5 Village, 6 Village, 7 Village, 9 Village and 11 Village, respectively (Fig. 1), aged 4, 13, 27, 28, 42 and 47 years, and 4 were males (4/6, 66.7%). Five patients developed the disease in late August and one in mid-October. The clinical manifestations of the cases were mainly fever and accompanying symptoms, including headache 5 of 6 (83%), fatigue 6 of 6 (100%), myalgia 1 of 6 (17%), all of which were high fever; gastrointestinal symptoms including nausea 3 of 6 (50%), vomiting 2 of 6 (33%), and diarrhea 3 of 6 (50%) were also prevalent, and five cases showed at least one gastrointestinal symptom; none had neurological manifestations (Table 3).

**Table 3:** Information of 6 WNF cases in Kashgar Region, 2013.

Number	Gender	Age (years)	Address	Illness onset	Symptoms										
					Fever (°C)	Headache	Asthenia	Nausea	Vomit	Diarrhea	Myalgia	Joints pain	Lymph node swelling	Skin rash	Nerve system^a^
1	Female	13	5 Village	20130819	40.5	√	√	√	√	√					
2	Female	27	6 Village	20130822	39.0	√	√	√		√					
3	Male	28	11 Village	20130825	39.5		√				√				
4	Male	4	9 Village	20130831	39.0	√	√	√	√						
5	Male	42	6 Village	20130831	39.5	√	√								
6	Male	47	7 Village	20131011	39.0	√	√			√					
Total					6	5	6	3	2	3	1	0	0	0	0

^a^Neurological manifestations include: unconsciousness, lethargy, coma, paralysis, paralysis, pathological reflexes, convulsions, nuchal rigidity, tremor, muscle w weakness, asymmetrical deep tendon reflex retardation or disappearance, facial nerve paralysis, etc.

### Animal WNV infection

In 2013, blood samples of domestic chickens were collected consecutively from 6 Village, 7 Village, 8 Village, and 9 Village in Jiashi County (2–3 times per chicken, with May–August sampling in 6, 7, and 8 Village and August–October sampling in 9 Village) since May 14. A total of 380 chicken blood samples were collected, of which 59 chicken sera were positive (≥ 1:10) for WNV neutralizing antibodies, with a total positive rate of 15.5%. The positive rate was 8% in May, suggesting that the local transmission of WNV had already occurred in May, followed by a gradual increase in the positive rate, which could last at least until October (Table 4).

**Table 4:** Results of neutralizing antibody against WNV in serum of chickens in Kashgar Region, 2013 (PRNT90).

Month	May			June			Auguest			October		
Address	Sample	Positive	Positive rate (%)	Sample	Positive	Positive rate (%)	Sample	Positive	Positive rate (%)	Sample	Positive	Positive rate (%)
6 Village	38	2	5.3	38	2	5.3	38	7	18.4	–	–	–
7 Village	36	4	11.1	36	5	13.9	36	6	16.7	–	–	–
8 Village	26	2	7.7	26	3	11.5	26	8	30.8	–	–	–
9 Village	–	–	–	–	–	–	40	6	15.0	40	13	32.5
Total	100	8	8.0	100	11	11.0	140	27	19.3	40	13	32.5

Chicken blood was not collected in July and September.

During the August–September of 2015, 167 animal (166 sheep and 1 cattle) serum samples were collected from the slaughterhouses in Kashgar Region. 3 sheep sera of WNV neutralizing antibody was positive (≥ 1:10) by PRNT90, and the positive rate was 1.78%.

### Mosquitoes

A total of 15,637 mosquitoes were collected in 2013–2016, with *Culex pipiens* (11,108) as the dominant mosquito species, accounting for 71% of the collected mosquito population (Table 5).

**Table 5:** WNV surveillance in mosquitoes in Kashgar Region, 2013–2016.

Year	*Culex pipiens*				*Aedes caspius*			
	Numbel of samples	Pools	WNV positive pools	MIR	Numbel of samples	Pools	WNV positive pools	MIR
2013	7837	156	4	0.051% (0.016%, 0.14%)	1373	27	0	0
2015	607	11	0	0	0	0	0	0
2016	2664	53	1	0.038% (0.002%, 0.24%)	3156	43	0	0
Total	11,108	220	5	0.045% (0.017%, 0.11%)	4529	70	0	0

A total of 9210 mosquitoes (7837 *Cx. pipiens* and 1373 *Ae. caspianus*) were collected in 2013. All samples were grouped in 183 pools (about 50 in each pool). Among the 183 mosquito pools tested for WNV RNA, 4 pools of *Cx. pipiens* collected in 7 Village (2 pool), 8 Village (1 pool) and 9 Village (1 pool) in Jiashi County were positive by PCR (Table 5), which were confirmed by nucleotide sequencing. The minimum infection rate (MIR) calculated for *Culex* was 0.051% (0.016%, 0.14%). The earliest WNV-positive pool was sampled in 9 Village on August 5, and the latest was sampled in 7 Village on September 3. In addition, supernatants of the 4 WNV-positive mosquito pools were inoculated onto Vero cells. One pool yielded 1 virus isolate. Phylogenetic analysis showed that the strain was Lineage 1 (data not shown).

A total of 607 mosquitoes were collected in 2015, all of which were *Cx. pipiens*. All 607 mosquito samples were grouped in 11 pools and none of these mosquito pools were WNV RNA-positive.

In 2016, a total of 6067 mosquitoes (2664 *Cx. pipiens* and 3156 *Ae. caspianus*) were collected and grouped in 96 pools. One pool of *Cx. pipiens* sampled on June 14 was found to be WNV weakly positive, and the MIR for *Culex* was 0.038% (0.002%, 0.24%). The supernatant of this mosquito pool was inoculated onto Vero cells and no WNV was isolated.

## Discussion

WNV originated in Africa and spread from there to Asia, Europe and the Americas. Disease in Asia is relatively rare, but the virus has emerged in Europe and the Americas, resulting in periodic large outbreaks. Because mosquito activity varies according to climatic conditions, it is difficult to predict where West Nile activity will occur, and because vertebrate hosts are birds, migratory birds have the potential to carry the virus for long-distance transmission. And humans are dead end hosts and unlike migrating birds cannot disseminate the virus to new areas. As global travel increases, the spread of West Nile infection among travelers will also increase.

During the August–September of 2004, an outbreak of viral meningitis and encephalitis caused by WNV occurred in Kashgar Region, Xinjiang China, although laboratory detection have demonstrated WNV circulation in humans^11,12^, as well as in mosquitoes^9^ in the years since the first WNV epidemic in 2004. However, to date, no information on the epidemiological characteristics of the WNV in the Kashgar Region has been available. Currently there is no WNV vaccine for humans; therefore, surveillance and control measures play a key role in the management of the spread of the disease. China began to monitor WNV disease every year in Kashgar Region, Xinjiang from 2013, and aim for early detection of WNV circulation and provides data needed for monitoring potential changes in disease transmission patterns and developing prevention strategies.

This study preliminarily elucidated the epidemiological characteristics and clinical manifestations of WNV disease in Kashgar Region for the first time. WNV infection in humans in Kashi Region shows a clear seasonal distribution, with an annual peak in August–September, which is consistent with the seasonal fluctuations of mosquito vectors^22^. There was a predominance of males with IgM positivity in all age groups, suggesting that men might be more frequently bitten by infected mosquitoes than females. In terms of confirmed cases, the majority of the six confirmed cases of WNV were found in young and middle-aged people, and there were two patients of young age (13 years and 4 years), with no confirmed cases and severe patients over 50 years of age, which was inconsistent with the epidemic characteristics in the United States^23^ (mostly middle-aged and elderly cases), and the reasons for this need to be further studied.

There was a big difference in the number of cases of WNV disease between 2013 (51) and 2016 (11), suggesting that local incidence of WNV disease may alternate between high and low incidence years. It was reported that a WNND outbreak occurred in 2004 in Jiashi County with many cases with high mortality^10^^.^ Six cases were laboratory confirmed in 2013, and there were no laboratory confirmed cases in 2014, 2015 and 2016, On the basis of our WNV IgM and neutralizing antibodies detection results from human samples, the WNV prevalence in Kashgar Region was low.

In this study, we found that WNV neutralizing antibodies appeared in chicken sera in May, suggesting that the emergence of WNV in local areas may be early, and the positive rate of infection in chickens is high, so it can be considered as an early indicator of the degree of WNV activity.

Accurate identification of mosquito species in an area is important to reveal and predict the emergence of WNV, because only certain species in any given area can effectively act as primary vectors and bridge vectors for human or equine populations^21^. Evidence from Italy and Spain suggests that its detection in mosquitoes precedes the appearance of human or equine cases arguing for its application in any surveillance strategy for WNV and other zoonotic arboviruses in areas at risk of incursion^24^. Another example provided by Turkey is that infected mosquitoes could be detected about 4 weeks before the emergence of human infection cases in the East Thrace region^25,26^. Our monitoring data indicates that the earliest time for nucleic acid positives in mosquitoes in 2013 was on August 5 and in 2016 was June 14. The earliest time for the WNV cases in 2013 was on August 19. So the time of mosquito nucleic acid positivity in Jiashi County was earlier than the time of occurrence of the cases. Thus, monitorization of WNV activity via vector surveillance is important in Jiashi County, China. *Cx. pipiens* mosquitoes are considered the most important vectors in transmitting WNV in Asia^27–29^. This study confirmed that *Cx. pipiens* is the main vector of WNV disease in Kashgar Region. Other mosquito species, such as *Ae. caspianus*, were not found to be infected with WNV.

WNV is typically a seasonal disease strongly associated with mosquito activity. Targeted surveillance for pathogens within mosquito populations offers the ability to detect viruses prior to their emergence in livestock, equine species or human populations^24^. Surveillance of WNV relies on multiple pillars. Laboratory testing of chickens, cattle and sheep, together with monitoring of mosquito pools during WNV epidemic season provide relevant information to public health authorities about the risk of human infections with WNV. Six confirmed WNV cases in 2013 along with the high infection rate of mosquitoes (MIR 0.51) and chickens (neutralizing antibodies positive rate 15.5%) in the same year well demonstrate remarkable activity of the virus. Likewise, there is no confirmed human infections correspond to the low infection rate of mosquitoes and sheep (neutralizing antibodies positive rate 1.78%) in 2015 also demonstrate that the virus activity was not very frequent.

Monitoring of WNV disease was continuously conducted in Kashgar Region of Xinjiang from 2013 to 2016. The monitoring data indicated that cases of WNV disease, including WNF and WNND, were present in Kashgar Region^11,12^, suggesting that WNV can persistently exist locally. WNV nucleic acids were detected and virus was isolated from the local dominant mosquito species (*C. pipiens*). Furthermore, evidence of WNV infection was observed in domestic animals such as chickens and sheep. From these data, we can confirm that Kashgar Region, especially Jiashi County, may be natural epidemic foci of WNV disease.

The persistence of WNV may lead to future outbreaks on bird migration routes in Xinjiang and between China and neighboring countries, which highlights the need for entomological studies on persistence mechanisms and identification of effective mosquitoes that act as bridges in WNV transmission^30^. Establishing avian surveillance based on serological surveys of poultry cohabiting with humans and/or sentinel chickens to assess animal transmission of WNV may provide useful epidemiological information, such as low WNV circulation, and thus can be used as an early warning system, as well as early detection of virus circulation to implement prevention and control measures^31,32^. Consequently, the trend highlights the routine virology surveillance in WNV surveillance cases, mosquitoes and avian should be maintained and enhanced in Kashgar Region of Xinjiang so as to prediction and early warning of outbreak an epidemic of WNV in China.
